# Solitary death and new lifestyles during and after COVID-19: wearable devices and public health ethics

**DOI:** 10.1186/s12910-021-00657-9

**Published:** 2021-07-10

**Authors:** Eisuke Nakazawa, Keiichiro Yamamoto, Alex John London, Akira Akabayashi

**Affiliations:** 1 Department of Biomedical Ethics, Faculty of Medicine, The University of Tokyo, 7-3-1 Hongo, Bunkyo-ku, Tokyo, 113-0033 Japan; 2 Office of Bioethics, Center for Clinical Sciences, National Center for Global Health and Medicine, Tokyo, Japan; 3 Department of Philosophy, Carnegie Mellon University, Pittsburgh, USA; 4 Division of Medical Ethics, New York University School of Medicine, New York, USA

**Keywords:** Solitary death, Public health, Ethics, COVID-19, Wearable device, Autonomy

## Abstract

**Background:**

Solitary death (*kodokushi*) has recently become recognized as a social issue in Japan. The social isolation of older people leads to death without dignity. With the outbreak of COVID-19, efforts to eliminate solitary death need to be adjusted in line with changes in lifestyle and accompanying changes in social structure. Health monitoring services that utilize wearable devices may contribute to this end. Our goals are to outline how wearable devices might be used to (1) detect emergency situations involving solitary older people and swiftly connect them with medical treatment, to (2) reduce the frequency of deaths that remain undiscovered and (3) to reduce social isolation by promoting social interaction.

**Methods:**

Theoretical and philosophical approaches were adopted to examine ethical issues surrounding the application of wearable devices and cloud-based information processing systems to prevent solitary death in the world with/after COVID-19.

**Main body:**

(1) Technology cannot replace social connections; without social support necessary to foster understanding of the benefits of health management through wearable devices among older adults, such devices may remain unused, or not used properly. (2) Maturity of the technology; systems face the difficult task of detecting and responding to a wide range of health conditions and life-threatening events in time to avert avoidable morbidity and mortality. (3) Autonomy and personhood; promoting the voluntary use of wearable devices that are a part of larger efforts to connect isolated individuals to a community or social services might be effective. Legal force should be avoided if possible. There is some concern that landlords may require an older person to sign a contract agreeing to wear a device. The autonomy of solitary older people should be respected. (4) Governance: policies must be developed to limit access to data from wearables and the purposes for which data can be used.

**Conclusion:**

If thoughtfully deployed under proper policy constraints, wearable devices offer a way to connect solitary older people to health services and could reduce cases of solitary death while respecting the personhood of the user.

## What is solitary death?

In Japan, deaths of isolation or solitary deaths, referred to as “*kodokushi*” or “*koritsushi*,” have recently been recognized as a social issue. While many definitions of solitary death have been presented [1, 2], its core features are deaths: (1) occurring at home, (2) involving someone living alone (3) under conditions of social isolation (no care, no constant contact, lack of social connections) [1, 2]. For the present discussion we are concerned with cases where (4) isolation is not the result of imprisonment or coercion and, (5) death is not the result of the isolated person taking their own life.

**Fig. 1:**
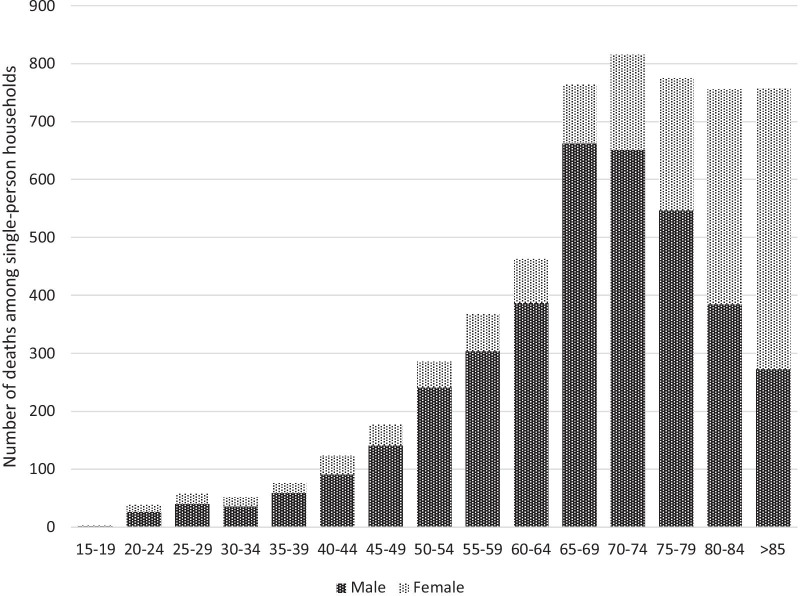
Number of at-home deaths in single-person households by gender and age group (2018). Shown is the number of unnatural deaths (other than deaths due to intrinsic diseases with reliable diagnoses) in single-person households in Tokyo, as reported by the Bureau of Social Welfare and Public Health (Tokyo Metropolitan Government) in 2018

Given the many definitions of solitary death, there are no accurate statistics on the number of solitary deaths in Japan [3]. But, for reference, a survey conducted among the 23 wards of Tokyo, Japan, revealed that in 2003, 1451 deaths among those aged 65 or older were of individuals living alone and who died in their own home [4]. By 2018, this number had increased to 3882 [5], suggesting that solitary deaths have been on the rise in recent years. The Fig. 1 shows data from the Bureau of Social Welfare and Public Health of the Tokyo Metropolitan Government [6]. These data reveal that, while older persons are not the sole demographic in which solitary deaths occur, these events frequently involve people 60 or older.

Solitary deaths are tragic for several reasons. Without family or friends present to detect warning signs of life-threatening events, older people living alone (hereafter, “solitary older people”) may die from preventable causes. Even if death is unavoidable, most individuals prefer not to die alone without the comfort or companionship of loved ones. Finally, it can take days or weeks to discover the body of a deceased person who passes in isolation. Such delays can be traumatic for family members of the deceased. They can also have negative public health consequences and adverse economic impacts on property owners.

According to surveys, the great majority of Japanese people feel that dying without caregivers attending at the deathbed (*mitori*) is “pitiful” (87%), and hope that someone will be present when they pass (62%) [7]. People fear the prospect of solitary death and regard it as an immediate concern [8]. These attitudes reflect common cultural beliefs among Japanese people regarding the importance of preserving human remains [9], and a conception of the life of a person that extends beyond the boundaries of physical death. In the latter view, markers of death, such as cessation of cardio-pulmonary functions and the decay of flesh, are regarded as events in the life of a person because individuals are viewed as socially situated and partly constituted by relationships and social bonds that persist after death. The biological death of a person is a momentous event within a life that persists among family and friends and in spaces where information about that person is shared and discussed until they fade from memory and finally case to exist. As a result, the manner of a person’s biological death influences the quality of a biographical life that is not yet over. Solitary death is regarded as particularly tragic for the person who dies alone because it is seen as an undignified transition within a life that is not fully over. It may also be seen as a tragedy and a failure by family, friends, and to some extent the larger community who bear the burden of this knowledge and in whose thoughts and stories the biologically dead live on.

## Solitary death and public health policy

The social isolation of older people is an important public health issue [10, 11]. Addressing it requires the implementation of measures to incorporate solitary older people into the local community. Some measures have been taken in Japan by local governments to prevent loneliness among older persons. Ideally, social isolation should be reduced by investing in programs that reconnect older people to social communities. For instance, in the short term, governments have been designing systems to link older people to community services [12]. In the longer term, revitalization of local communities is crucial for the social inclusion of older people. A list of such measures compiled by the Ministry of Health, Labour and Welfare (MHLW) includes: (1) surveillance and gaining an understanding of the current state of older people, (2) creating initiatives in collaboration with private businesses such as newspaper delivery companies, and (3) establishing a general consultation desk [13]. The emphasis of MHLW’s policy is to improve awareness and contact with older people, in part, by involving private sector actors with established infrastructure and large numbers of personnel that routinely circulate widely in the community, such as newspaper delivery companies, gas companies, and electric power companies. Because such personnel are not health services specialists and are not part of a single communication network, a central hub for reporting information and coordinating response is required. For example, in Tochigi Prefecture, the Isolated Death Prevention Surveillance Service (known as “*Tochimaru Watchover Net*”) was established as a system for reporting concerns to a common respondent, and represents a collaborative network of the prefectural office, the police, municipalities, local welfare officers, and utility suppliers (gas, water, and telecommunication) [14]. To give another example, in Iwate Prefecture, the monitoring system which uses information and communication technology (*Iwate Ogenki Mimamori System*) asks that older people in the system voluntarily and actively report their health status to the “watch system” by phone (i.e., “I’m fine,” “I’m a little tired,” “I feel sick,” and “I need some help”). The individual’s health status is reported to the monitoring center in the municipal social welfare council and to the family from the monitoring system [15, 16].

These local government initiatives represent ways to increase contact with older persons and are considered relatively effective. Ideal settings for implementing these measures are relatively rural areas, where local communities are already fairly well formed. Many examples of similar measures implemented in rural areas have been reported in Japan [13]. The bigger issue is social isolation of older people in urban areas, where local communities are not as close-knit. Upstream issues underlying social isolation of older people in urban areas include the rapidly declining birthrate, an increased number of single individuals, and the increase in social disparities. As is the case for other public health interventions, we must consider the upstream causes and potential interventions to address the downstream effects; namely, the prevalence of solitary deaths.

## COVID-19 and the risk of solitary death

The outbreak of COVID-19 and associated public health responses has the potential to exacerbate dynamics that result in solitary deaths. Mortality rates from the virus increase precipitously with age. In the absence of a vaccine, reducing physical contact and proximity plays an important role in safeguarding the health of the older people, while potentially increasing social isolation. The importance of being able to monitor the health of solitary older people and to provide timely social and medical assistance thus increases in the pandemic context.

Since March 2020, when restrictions were placed on citizens’ lives, numerous cases of solitary death related to COVID-19 have been reported. Cases of solitary death during the pandemic include suspected COVID-19 patients with mild symptoms who died at home or in hotels after their conditions worsened rapidly during observation [17, 18]. While reducing the number of solitary deaths directly caused by COVID-19 is a pressing task, consideration must also be given to the social issue of solitary death caused indirectly by COVID-19 over the longer term. The Japanese government announced the “new lifestyle” in May 2020, implying a behavioral transformation that assumes a situation in which society is chronically threatened by COVID-19. The “new lifestyle” entails social distancing, wearing a mask, and washing hands as basic measures against infection [19, 20]. These measures were later incorporated into the WHO’s “Avoid the Three Cs” call [21]. It also includes recommendations to restrict people’s movements, such as refraining from going out, promoting telework, and recommending online meetings. The “new lifestyle” is expected to curb the further spread of COVID-19 in Japan, but is also likely to increase the isolation of older persons.

The development of several safe and highly effective vaccines for COVID-19 offers the prospect that some elements of the “new lifestyle” may be temporary. However, the challenges of achieving widespread vaccine coverage across the globe and emerging variants of the SARS-CoV2 that are more infectious may result in the continuance of practices and policies that contribute to social isolation among the elderly into the foreseeable future. Such policies and practices may contribute to the incidence of solitary death as a result. For instance, social distancing has led to physical and psychological isolation of the older people. Public health measures that reduce travel prevent relatives from visiting older family members during medical emergencies and there are increasing reports of incidents in which, in order to avoid COVID-19, family members living far away are unable to claim the bodies of older people living alone. Instead, remains are cremated without family visitation [22]. If this is regarded as a specific manifestation of the “new style of death,” then action must be taken to reduce these tragic deaths.

## COVID-19 and health information: wearable devices

Solitary death has been a social problem since before the COVID-19 pandemic. In society with/after COVID-19, it will be important to adjust efforts to eliminate solitary death in line with changes in lifestyle and accompanying changes in social structure. The main pillars of efforts to address the problem of solitary death before the COVID-19 pandemic were local community-based monitoring services. These services are highly important because they not only monitor the health of older persons, but also provide them with social capital from the local community. Yet, community-based monitoring services face problems of feasibility in urban areas, due to, for example, the decline in local communities in Japan’s cities, a major problem for more than 20 years [23]. Even in rural areas, where such monitoring services for older adults have been most effective, some note struggles to sustain the bare minimum required of a community due to the rapidly decreasing birthrate and aging population [24]. Such circumstances have strained the ability of local communities to provide social capital. The demands for social distancing with/after COVID-19 will greatly increase the difficulty of implementing community-based monitoring services for older adults. Thus, novel approaches will be needed to eliminate solitary death in the context of new lifestyles with/after COVID-19.

As wearable devices increase in affordability, availability and functionality, they are likely to play a greater role in health monitoring services for older adults. A wearable device is a downstream intervention that can connect solitary older people with medical and emergency services. Wearable medical alert systems have been available for decades and have allowed the older people to call emergency services for help. However, such systems had to be activated by the person themselves. Wearable devices paired with cloud-based information processing systems open the possibility of expanded functionality such as the ability to monitoring the health of the user and to transmit a broader bandwidth of data to designated recipients in circumstances where the user is incapacitated. Data from continuous monitoring can be transmitted human personnel, such as designated nurse stations for analysis and alerts could be sent to emergency response teams and to designated family or friends.

More ambitious approaches might seek to automate larger portions of this process [25–29]. In that case, information from wearable devices might be an input into systems that use artificial intelligence or machine learning to monitor aspects of a person’s health, build a model of their routine functioning or their mental health [30] and attempt to predict life-threatening events before they occur. If techniques can be developed to detect life-threatening events with sufficient sensitivity and specificity, the swift dispatch of emergency medical assistance may reduce rates of avoidable death. Even if fatal conditions cannot be detected with sufficient lead time to permit life-saving intervention, the prompt detection of and response to signs of life-threatening events could reduce the number of situations in which the passing of a solitary older person goes unnoticed. Emergency services could be notified of a person’s passing in a timely manner, reducing delays in the discovery of remains and the notification of loved ones.

Currently, various wearable devices are being put into practical use, and research and development are ongoing. In January 2019, the first wristwatch-type blood pressure monitoring smartwatch with Bluetooth technology to receive FDA approval, was released by Omron [31, 32]. ECG monitoring and fall detection capability had already been introduced by Apple in 2018, with the release of the new smart watch, which can detect (‘self-check’) an irregular heart rate [33]. Empatica’s wristband can monitor body temperature and movement data via an axis accelerometer, measure pulse rate using a photoplethysmogram and psychogenic sweating through electrodermal activity [34]. This wearable device is linked to a cloud-based information processing systems that can be paired with additional wearable devices to detect and alert users to the onset of epileptic seizures [35]. Similar systems are being used to provide alerts about COVID-19 infection risk [36].

A research team led by Associate Professor Kuniharu Takei at the Osaka Prefecture University Graduate School of Engineering developed the prototype of a printed, soft wearable device that can be attached to the body like a bandage. This “bandage” allows for measurement of heart rate, skin temperature, UV exposure level, and activity level by simply attaching it to one’s body [37].

These types of wearable devices for health information management are unique in that they are non-invasive, easy to wear and remove, simple to operate, and do not restrict other actions. In addition, if the devices are equipped with a telecommunication capability to allow access to the Internet (e.g., the Apple Watch Series 4), not only the automatic transmission of data, but the management and analysis of data in the Cloud also become possible.

Remote sensing of health data from older users and cloud-based information processing systems are an active area of research with projects seeking to provide a wide array of support, from predicting and preventing halls or disease specific events to supporting the mental health of older adults. To the extent that such systems can avert accident, injury or disease related morbidity and mortality they hold out the prospect of helping to reduce the prevalence of isolated death. The imminent causes of death can be predicted, such systems hold out the prospect of notifying loved ones or emergency services in time to avoid passing in isolation with death cannot be averted. Finally, in the event of sudden demise, such systems hold out the prospect of being able to notify loved ones or emergency personnel in a timely manner in order to avoid lengthy delays in discovering the deceased.

## Challenges of wearable devices

Some key challenges facing efforts to use wearable devices to avert deaths of isolation can be usefully grouped under 4 general headings: (1) technology cannot replace social connections, (2) the maturity of the technology, (3) autonomy and personhood, and (4) governance.

### Technology cannot replace social connections

Japanese cultural beliefs about the boundaries of life and death foreground the extent to which death is an event that takes place within the biographical life of a person who is connected to others through bonds of kinship, friendship, and community membership. This perspective usefully highlights and renders salient concerns that are likely shared across cultures, namely, that familial relationships, friendships, and social connections are widely regarded as important goods that enrich the quality of life and that being cut off and isolated from such connections likely to be distressing for older adults and their loved ones generally, not simply at the end of life. Social efforts to reduce the frequency of deaths of isolation should thus focus on creating opportunities for older adults to establishing and maintain meaningful social connections across the lifespan and not simply at the end of life. Technologies for monitoring the health and welfare of older adults should be understood and deployed as part of a larger, multi-faceted effort to provide social support to isolated older adults.

Although wearable devices represent a promising avenue for promoting the health interests of isolated older adults, they are not a panacea. Paradoxically, perhaps, although advances in sensor technology and machine learning might improve the sensitivity and specificity of devices, their success depends on a range of analog efforts by local communities to support isolated adults. First older older adults must be willing and able to use and to properly maintain such devices. This may require training and instruction. Second, accurately predicting or detecting critical events is of little value if that information does not propagate through a health or social services system that is designed to evaluate its credibility and to deploy proper services in a timely manner. On the technology side, this requires an infrastructure in which devices communicate with social services which are themselves connected in a communication network that can be sustained over time.

On the service provision side, this requires the development of protocols that incorporate signals from wearables in a way that monitors and respond properly to alerts that are true positives, false positives, and that can be adjusted to reduce the rates of false negatives. A false positive occurs when an alert or alarm is triggered even though there is no problem with the health of an older person. False positives can occur for a variety of reasons, including misuse of the device, device failure, or due to errors in classification (e.g., if an excited arm movement is classified as a fall). If a system produces too many false positives, then (a) resources may be expended needlessly in unnecessary responses or (b) stakeholders may lose trust in the signal and not respond.

Whether false positive results have a negative effect on users and systems will depend, in part, on the responses that alerts produce. If alerts produce responses that users regard as annoying, such as alarms or automated phone calls, false positives may reduce system utilization and therefor degrade efficacy. But if false alerts produce social interactions that users may value—such as a video call from a case worker with whom the user has a prior relationship—higher rates of false positives might not reduce the value of the system to users.

A false negative occurs when no alert is issued even though the older person is experiencing a health issue. False negatives can occur when an event happens that the device was not developed or trained to detect, when poor communication conditions lead to the data not being sent from the device, or when the device fails to operate properly or the device is improperly used, removed intentionally or unintentionally, or powered off. Since the accuracy of a device depends on the willingness and ability of the user to maintain it and operate it properly, wearable devices and systems that incorporate wearables must be designed in ways that give users a reason to faithfully wear and maintain them. This includes enhancing the operability, removability, comfort and the overall aesthetics of the device. More fundamentally, it may also involve linking device usage to activities that users’ value.

For example, studies have shown that exercise among older persons reduces the probability of falling and therefore of fall-related injuries [38]. Some users may value wearables with more interactive functionalities (such as smart watches) that encourage movement or exercise, track progress on such goals, and offer the ability to display photos or play music. In contrast, other users may find these extra capacities daunting and prefer wearables that are specifically designed to monitor particular health conditions. Based on the results of a previous study which found that, in addition to the cognitive dimension of perceived benefits and external influences, such as caregivers’ encouragement, elements of design and the minimization of disruptions to existing routines also influenced perceptions of wearing the devices [39], it can be inferred that matching user preferences to device functionality and attending to the way users experience false positives can increase compliance and user satisfaction.

Without social support necessary to foster understanding of the benefits of health management through wearable devices among older adults, such devices may remain unused, or not used properly. Helping users to remember to wear such devices, and promoting maintenance of the devices to avoid battery drainage or breakdown, cannot be the responsibility of older users alone. To create the conditions under which wearable devices can advance the health needs of older adults, communities must find ways to increase social supports that older adults need to be able to use and benefit from such devices.

### Maturity of the technology

Systems that seek to detect and responding to life-threatening events in time to avert avoidable morbidity and mortality have potentially significant social value, but they face the difficult task of detecting and responding to a wide range of health conditions. Although monitoring technologies are being developed for a wide range of purposes, a recent systematic review of smart home and home monitoring systems notes that although some technologies have advanced past the proof of concept stage, the “level of technology readiness for smart homes and home health- monitoring technologies is still low” [40]. Systems that are designed solely to detect acute events, including cardiac arrest or the cessation of cardiopulmonary activity may be more narrowly focused, but at the cost of potentially reducing their social value. The willingness of older adults to implement monitoring systems depends on the degree to which they see those purposes as supporting important goals, including preserving their ability to remain at home and to maintain an adequate quality of life [40–42]. It is unclear whether older adults would find sufficient value in a system that is less likely to avert their death but that might reduce the prospect that their passing will remain unnoticed. Moreover, such attitudes may differ across and within social and cultural groups.

### Autonomy and personhood

Incorporating wearable devices into a larger program of social support is also critical to using these devices on terms that respect the autonomy and personhood of users. Ideally, policy makers and public health officials should strive to create an environment in which solitary older adults proactively wear such devices because they perceive the benefits that they offer as outweighing any inconvenience or perceived risks. Promoting the voluntary use of wearable devices will require targeted research in the fields of design and human–computer interaction in order to determine how to configure such technologies so that they are attractive to users and help bridge important social connections. But it will also require a reconfiguration of social services to provide education, training and social supports to facilitate their voluntary and effective use.

Incentives such as rebate programs, discounts, or other forms of monetary or social reward may have a valuable role to play in encouraging or “nudging” uptake and use. But the use of such incentives should be carefully tailored to reduce barriers to effective usage without making usage a condition for accessing services or resources to which older adults are already entitled. They must also reflect the diversity among older adults, including the fact that older adults who live in poverty may have greater difficulty participating in such programs without assistance. Advertising campaigns or community building efforts that use social media may similarly exclude isolated adults who live in poverty. Therefore, finding appropriate methods to encourage participation in such programs will require ingenuity and multiple methods.

The use of punitive incentives to enforce the use of wearables among isolated older adults, including legal or contractual requirements as a condition of leasing property, should be avoided. First, punitive measures are not likely to address the hurdles to effective use facing many older adults including lack of familiarity with and technical ability necessary to properly use and maintain such devices. This is likely to hamper the effectiveness of the resulting system and to the extent that compliance failures are associated with social inequalities, is likely to produce the worst outcomes for the most socially vulnerable.

Second, legal or contractual requirements the use of wearables promote adversarial relationships in which intrusive monitoring is instituted to advance the interests of more advantaged parties—such as property owners seeking to preserve the value of their assets—without adequate regard for the autonomy, privacy and personhood of individuals in an already vulnerable population. Although social isolation is often an undesirable state for older adults, intrusive monitoring reduces a person’s legitimate interest in privacy, understood as the ability to retain control over one’s sensitive personal information, without increasing social inclusion. In fact, creating adversarial relationships with property owners, police or social services can increase the isolation of older adults.

Finally, the considerations outlined above reflect the importance of assessing the use of wearable devices from a standpoint that is broader than a narrow focus on autonomous decision making. In part, this is because the ability of individuals to make decisions that reflect their considered values depends on their ability to live in conditions that support their mental and physical health. Promoting autonomous decision making without attention to the material needs, mental and physical health of isolated individuals may not be sufficient to support the real freedom of those individuals to advance their fundamental interests.

To promote the real freedom of isolated individuals, wearable technologies must be one element within a multi-sectoral public health intervention aimed at supporting the mental and physical health of isolated individuals and at reducing social disparities that cause and perpetuate social isolation. Within this broader context, wearable devices might play a valuable role in connecting isolated older adults to social services, including public health and health care resources. Outside of such a context they may be ineffectual or potent tools for intrusive monitoring, manipulation and abuse.

### Governance

Implementing a program of health management for solitary older people using wearable devices will require a thoughtful governance system. Problems related to governance include issues about the accessibility of data (who will have access to the data), the purposes for which data can be used, and the provision of information to the older people.

From the standpoint of protecting privacy, health data obtained from wearable devices are highly sensitive. The purposes of using such data should therefore be limited to the objectives of reducing isolation among the older people, improving their health status, and eliminating solitary death. Even if data were to be handled by public institutions such as central and local governments, any use of the data that does not benefit older people must be restricted. Particularly in situations where conflicts of interest, albeit latent, exist between the government and citizens (e.g., data related to tax collection or information about the political thinking or beliefs of citizens), the facile linking of data with individuals must be strictly prohibited.

Potential providers of health management systems that use wearable devices include central and local governments, as well as public health centers and welfare councils that make up health and welfare networks. But such models often envision close partnerships between such networks and medical institutions, sometimes with inputs from private parties outside of healthcare. Personal information entered in such systems should be protected in accordance with confidentiality obligations of medical contracts, and central and local governments have a responsibility to maintain secure servers. While insurance and real estate companies could also build networks, they likely are not suitable candidates for constructing these systems when the sensitive nature of the data is taken into account.

Being conscious of the vulnerability of older people and protecting their privacy are intimately linked to respect for their autonomy. Naturally, the incidence of symptoms of dementia among older adults increases with age. Appropriate medical services and nursing-care services will be needed to prevent the isolation of older people with dementia. Wearable devices might have a valuable role to play in such systems, but such devices are not a substitute for them. For example, wearable devices with GPS function might be useful in addressing the issue of wandering among dementia patients, but only if that monitoring feeds into a larger ecosystem of health services that provide appropriate support for people living with dementia, including proper security for the privacy of such sensitive personal information.

On the other hand, governance system should be crafted to support the use of data, within the boundaries of proper safeguards, for epidemiological research by health authorities, universities, and other research institutions. The rationale for permitting research on such data is to advance the science necessary to improve our ability to meet the distinctive health needs of a population that is often underserved. Such an oversight mechanism should include provisions for making de-identified data available for only research projects aimed at promoting the well-being of older adults and should specify the conditions under which broad versus specific consent of users is required.

An important component of the governance of health management systems which handle health information concerns what information is provided and how that information is provided. As for the information being provided, it is essential to offer information on the risks of wearable device-based health management systems. Although it is unlikely that the physical risks of using most wearable devices will be high, there are information security risks that must be considered. Attention must be given to how information is provided, and specifically the usability of that information, given that the system’s users are older adults. Also important is that the provision of information does not occur merely in form alone.

## The potential of wearable devices and cloud-based information processing systems—eliminating isolation of older persons

It is possible that merely monitoring the health status of isolated older adults will not bring about a reduction in their social isolation. For this reason, wearable devices and cloud-based information processing systems should serve as upstream interventions that will not only help to link individuals to services and avoid solitary death, but also present a solution to the problem of isolation of older people in a way that contributes to the recovery and maintenance of their health. The new lifestyles brought about by the spread of COVID-19 pose fundamental challenges to this process.

Although it may only be for a limited time, the spread of COVID-19 had led to a significant easing of restrictions on online medical treatment in Japan and other countries [19]. Telemedicine holds the promise of enabling new types of relationships between healthcare providers and patients that transcend the limits of local infrastructure by expanding into virtual spaces. This presents the possibility of bringing medical consultations and some forms of treatment to isolated older adults with greater flexibility than ever before. Linkages between online primary care services and wearable device-based health management systems might offer a pathway to better incorporate solitary older people into the medical system. This includes linking older adults to primary care physicians, and a nexus of social support services.

But such linkages will require considerable effort, including social efforts to overcome the digital divide. Many older adults are unable to use the technologies necessary to receive medical care online. According to an NHS England survey of users of a smartphone app-based medical consultation service (GP at Hand), use was most prominent among people in their 20 s to 40 s, with only a handful of older users [43]. Once again, the paradox returns: in order to promote online medical care, it will be important to create an environment where isolated older adults receive the support necessary to get online and access social support services that are available there. Given the pressing need to pursue the potential of communication through means other than face-to-face contact, like SNS, innovations to include older people in such forms of communication are a must.

## Strengths, limitations, and further perspectives

Social isolation and solitary death are important public health issues that are exacerbated by the new lifestyle adopted during the COVID-19 pandemic. COVID-19 has brought about enormous changes in the lifestyle and medical settings inhabited by older persons. This impact will not be easily undone even if vaccinations become more common. Rather, it is likely that COVID-19 will have lasting societal after-effects, with isolation among older persons being one. The strengths of this study include the following: 1) it considers the problem of solitary death occurring among older persons in lifestyle and medical environments radically altered by COVID-19, and 2) it explores the utility of wearable devices with respect to medical care settings after/with COVID-19. Furthermore, we discuss ethical issues from three perspectives: (1) technology cannot replace social connections, (2) the maturity of the technology (3) autonomy and personhood and (4) governance. Until now, no study has explored these areas. We hope that our research will mark a first step spurring wider engagement with this problem.

We recognize, however, that our discussion has certain limitations. The first is the issue of generalizability. We identified solitary death in Japan as a social problem in an after/with COVID-19 context. Society’s rapid aging, the expansion of relative poverty, strained medical systems for older persons, and the weakening of family or community networks are among factors exacerbating the problem of solitary death in Japan. Although the phenomenon of isolation among older persons is ubiquitous around the world, it is likely that different countries or communities will face distinct problems in attempting to address this issue.

Second, we have focused on issues that are likely to apply generally to systems that use wearables to address the problem of deaths of isolation. We recognize that specific examples of such systems may raise a spectrum of additional ethical issues. For example, wearables that use green light to detect heart rate or blood oxygen levels may not function with equal efficacy or accuracy across individuals with darker and lighter skin tones [44–46]. The use of such systems raises important issues of equity and fairness. Similarly, health systems are organized on different principles in different nations and these differences can pose challenges for the equitable implementation of systems that are complex, expensive, and require coordinated efforts on the part of health, social services, or other elements of civil society. Because aging, cognitive and physical decline, death and the value of familial and social connections are universal human experiences, care must be taken to ensure that systems function equitably across social and economic groups.

Our hope is that the present discussion is sufficient to spark futher interest in this topic frm specialists in a wide range of disciplines. This includes further social science research to investigate the attitude of older adults toward the use of wearables specifically to address aspects of isolated death. It also includes further work at the intersection of science and technology studies and ethics of AI about the particular challenges posed by specific platforms that might be deployed in this context.

## Conclusion

Wearable devices provide hold promise as a promising platform for reducing solitary deaths and for linking socially isolated individuals to a broader range of health and social services. But these technologies cannot be effectively deployed in these populations on terms that respect older persons without considerable social support. This support includes closing the digital divide, tailoring devices to user preferences, supporting the ability of users to generate value from these devices and ensuring that there is proper governance of the information they produce. Although such devices are not a panacea, their ability to connect isolated individuals to a wide range of services is a worthy target for public health measures aimed at supporting this underserved population.

The social implementation of new technologies invariably brings certain costs. For wearable devices, not only economic but also labor costs will be incurred. Yet, we believe this will contribute to envisioning a better society. In order to avoid isolation among older persons, and alleviate the problem of solitary death even marginally, our society must proactively explore ways of providing public health support. Wearable devices represent one promising tool for achieving this.

## Data Availability

Not applicable.

## Abbreviations

COVID-19: Coronavirus Disease 2019
ECG: Electrocardiogram
FDA: U.S. Food and Drug Administration
MHLW: Ministry of Health, Labour and Welfare
NHS: National Health Service
